# Gram‐Scale Synthesis of 41% Efficient Single‐Component White‐Light‐Emissive Carbonized Polymer Dots with Hybrid Fluorescence/Phosphorescence for White Light‐Emitting Diodes

**DOI:** 10.1002/advs.201902688

**Published:** 2020-01-16

**Authors:** Zifei Wang, Yang Liu, Shijie Zhen, Xiaoxi Li, Weiguang Zhang, Xun Sun, Baoyuan Xu, Xue Wang, Zhenhua Gao, Xiangeng Meng

**Affiliations:** ^1^ School of Materials Science & Engineering Qilu University of Technology (Shandong Academy of Sciences) Jinan 250300 China; ^2^ State Key Laboratory of Luminescent Materials and Devices South China University of Technology Guangzhou 510640 China

**Keywords:** carbonized polymer dots, high quantum yield, hybrid fluorescence/phosphorescence, white light emission, white light‐emitting diodes

## Abstract

Fluorescent carbon dots (CDs) are compelling optical emitters to construct white light‐emitting diodes (WLEDs). However, it remains a challenge to achieve large‐scale and highly efficient single‐component white‐light‐emissive CDs suitable for WLED applications. Herein, a low cost, fast processable, environmentally friendly, and one‐step synthetic approach is developed for the preparation of gram‐scale and highly efficient single‐component white‐light‐emissive carbonized polymer dots (SW‐CPDs). It is revealed that hybrid fluorescence/phosphorescence components cooperatively contribute to the emergence of white light emission. The SW‐CPDs exhibit a record quantum yield (QY) of ≈41% for the white light emission observed in solid‐state CD systems, while the QY of the phosphorescence is ≈23% under ambient conditions. Heavy doping of N and P elements as well as presence of covalently cross‐linked polymer frameworks is suggested to account for the emergence of hybrid fluorescence/phosphorescence, which is supported by the experimental results and theoretical calculations. A WLED is fabricated by applying the SW‐CPDs on an UV‐LED chip, showing favorable white‐light‐emitting characteristics with a high luminous efficacy of 18.7 lm W^−1^ that is comparable to that of state‐of‐the‐art WLEDs reported before.

## Graphical abstract

Gram‐scale single‐component white‐light‐emissive carbonized polymer dots (SW‐CPDs) containing two emission centers of blue fluorescence and green room‐temperature phosphorescence with an overall quantum yield up to 41% are successfully prepared. A white light‐emitting diode (WLED) is fabricated by employing the SW‐CPDs with a commercial UV‐LED chip. This work provides an important step toward development of high‐performance CPDs‐based WLEDs.

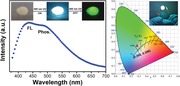

White light‐emitting diodes (WLEDs) have become a strong contender as next‐generation solid‐state lighting sources owing to their advantages of energy conservation, long lifetime, and high luminous efficacy.^1^, ^2^ Most WLEDs developed thus far are fabricated by combining multicomponent light emitters with different emission colors covering the entire visible range from 400 to 700 nm.^3^, ^4^ However, the resultant WLEDs usually suffer from a series of issues, such as self‐absorption, phase separation, color‐aging, fabrication complexity, poor stability, reproducibility, etc.^5^ Recent studies suggest that single‐component white light emitters could potentially overcome the above issues.^6^, ^7^ Therefore, it is of great significance to explore single‐component materials that can directly serve as white light emitters. In principle, such materials can be reached by rationally designing multiple light‐emitting centers in a single material system. For instance, single‐component white‐light‐emitting materials can be realized with two co‐existing emissive centers, where the short wavelength‐emissive center is created by the lowest singlet excited state while the long‐wavelength center can be generated by various excited states such as charge‐transfer state,^8^ excimer state,^9^ excited‐state intramolecular proton transfer systems,^10^ conformation‐dependent emission systems,^11^ and triplet excitonic states for phosphorescence.^12^ Among the above‐mentioned emissive centers, phosphorescence process has attracted growing interest for the development of WLED devices due to high energy conversion efficiency and low heat value caused by sufficient utilization of the triplet state.^13^ However, single‐component white light‐emitting materials showing fluorescence/phosphorescence dual emissions reported so far are rather limited, which are mainly restrained to be materials based on conventional rare‐earth ions, organometallic complexes, and metal‐free organic compounds.^14^, ^15^ Unfortunately, these materials usually suffer from high cost, toxicity, low thermal and photostability, sensitivity to ambient atmosphere, and complicated fabrication processes. Thus, it is in high demand to call for development of novel single‐component white‐light‐emitting materials that merit in low cost, nontoxic, fast processable, excellent thermal and photostability, and large‐scaled fabrication.

Carbon dots (CDs) have emerged as a new class of luminescent nanomaterials exhibiting tunable fluorescence (FL), high thermal/photostability, low cost, and environment‐friendliness, thus holding potential applications in a variety of areas.^16^, ^17^, ^18^, ^19^, ^20^, ^21^ Recently, FL/phosphorescence dual emissive properties have been discovered in CDs, which have been demonstrated as promising phosphors for WLEDs.^22^, ^23^ However, all of these CDs obtained thus far have been limited to low quantum yield (QY, < 25%) due to relatively poor phosphorescence emission, worse still, with extremely low synthetic yield. Therefore, developing large‐scale and highly efficient single‐component white‐light‐emissive CDs with FL/phosphorescence dual emissions for achieving high‐performance WLEDs is highly desired.

Carbonized polymer dots (CPDs), as a new type of fluorescent CDs, inherit the advantages of both facets of composite‐based CDs materials, possessing both the excellent luminescent properties of CDs as well as the matrix effects imbued by polymers, which can substitute covalent bonds for supramolecular interactions and thus greatly enhance the fixation.^24^ Therefore, CPDs with abundant highly crosslinked network structure of nonconjugated groups offer an alternative, novel, and promising approach to achieving the efficient phosphorescence through an convenient synthesis approach.^25^ Here, we report on the fabrication of gram‐scale single‐component white light‐emissive CPDs (SW‐CPDs) with dual emission bands composed of blue FL and green phosphorescence via a one‐step heat treatment process of urea and phosphoric acid aqueous solutions (**Figure** **1**a). The obtained SW‐CPDs exhibit a record white light emission QY of ≈41% and relatively high phosphorescence with QY of 23% under ambient conditions. Systematic optical and structural characterizations as well as elaborate theoretical calculations suggest that the strategically designed covalently crosslinked polymer frameworks with heavy doping of N and P elements are the keys to observing strong white light emission. The FL and phosphorescence originate from the certain sub‐fluorophores containing N and P elements and n→π* electron transitions because of the small energy gap between first singlet state (S_1_) and first triplet state (T_1_), respectively. Combining the SW‐CPDs with an UV‐LED chip, we have realized a SW‐CPDs‐based WLED with Commission Internationale de I'Éclairage (CIE) coordinate (0.268, 0.346). A high luminous efficacy of 18.7 lm W^−1^ at a 20 mA drive current has been achieved in the resultant WLED, which is comparable to some WLEDs based on rare‐earth phosphors. The WLED based on SW‐CPDs shows high stability within a test period of 144 h.

**Figure 1:**
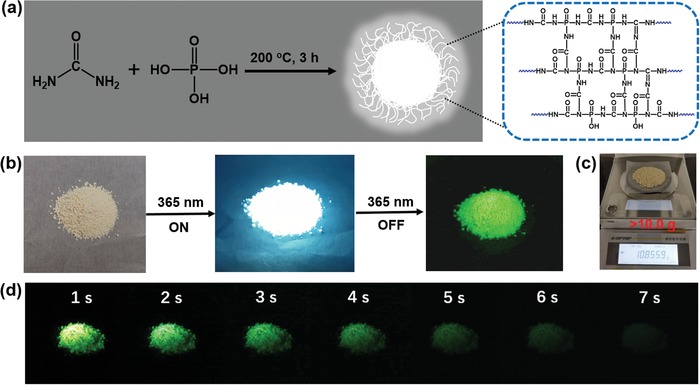
a) Schematic diagram showing the preparation and growth mechanism of SW‐CPDs. b) Photographs of SW‐CPDs captured under daylight (left), 365 nm UV lamp illumination (middle), and removal of the 365 nm UV lamp (right), respectively. c) SW‐CPDs obtained in a single reaction with weight exceeding 10 g. d) Images of SW‐CD powders after ceasing the UV lamp (365 nm) illumination for 1–7 s under ambient conditions.

In brief, a solution of urea and phosphoric acid is heated at 200 °C for 3 h, followed by rinsing with deionized water three times and subsequent freeze‐drying to obtain SW‐CPDs powders (see details in the Experimental Section). The obtained SW‐CPDs appear as pale yellow powders (Figure 1b), emitting bright white light with a QY of 41% under UV lamp (365 nm) irradiation (Figure 1b). Intense green phosphorescence with a QY of 23% is obtained after UV irradiation, which lasts for about 7 s (Figure 1b,d). The SW‐CPDs have a high production yield up to ≈81% (Table S1, Supporting Information) and gram‐scale (>10 g) production can be readily achieved in a single reaction conducted in a 50 mL beaker (Figure 1c), suggesting that the one‐step simple heating process is suitable for industrial‐scale production. As seen in **Figure** **2**a, the optical absorption spectrum of the SW‐CPDs exhibits an obvious band at around 280 nm together with broad‐band absorption about 380 nm, which may be attributed to the π–π* and n–π* transitions of certain sub‐fluorophores.^26^ The photoluminescence (PL) spectrum appears as a broad band with two distinguishable sub‐peaks located at ≈436 and 495 nm, respectively (Figure 2a). The broad‐band nature of the PL spectrum covering the entire visible spectral window suggests that such SW‐CPDs are advantageous for generating white light with high color rendering index (CRI). The lifetimes of the PL signals at ≈436 and 495 nm are estimated to be ≈3.96 ns and 320 ms, respectively, suggesting the FL and phosphorescence nature of these two bands (Figure 2b,c). Furthermore, these two different emissive components (FL and phosphorescence) can be confirmed by the time‐dependent PL spectra (Figure S1, Supporting Information). At a decay time of 160 ns, the FL component of SW‐CPDs disappears completely, while phosphorescence remains intense. The PL excitation (PLE) spectrum recorded by monitoring the PL peak at ≈436 nm shows a maximum at ≈375 nm, in good agreement with the absorption shoulder located at ≈380 nm. The PL signal located at 495 nm is further verified to be phosphorescence by comparing the phosphorescence spectra recorded at 298 and 77 K (Figure 2d). The PL signal of the SW‐CPDs shows rather slight degradation (<5%) under continuous illumination with a λ = 365 nm UV lamp (≈6 W) for 20 h (Figure 2e). The SW‐CPDs can be dispersed in water to form a clear solution under daylight (Figure S2a, Supporting Information). Bright white light can be observed when the solution is irradiated with a 365 nm UV lamp (Figure S2b, Supporting Information). The PL spectral profile of the SW‐CPDs solution is almost identical to that of solid‐state SW‐CPDs (Figure S3, Supporting Information), suggesting that the PL signal is originated from single CPDs instead of aggregates. The phosphorescence intensity of the SW‐CPDs immersed in water is nearly unchanged with respect to that of dry SW‐CPDs, indicating the high stability of SW‐CPDs in water (Video S1, Supporting Information). From the PL and phosphorescence spectra recorded at cryogenic temperatures (Figure S4 in the Supporting Information), the Δ*E*_ST_ value between S_1_ and T_1_ is calculated to be 0.09 eV (Figure 2f). To the best of our knowledge, the Δ*E*_ST_ value of the SW‐CPDs is the lowest for phosphorescence CDs and such a small value benefits in an effective intersystem crossing (ISC) process to populate triplet excitons.^25^, ^27^

**Figure 2:**
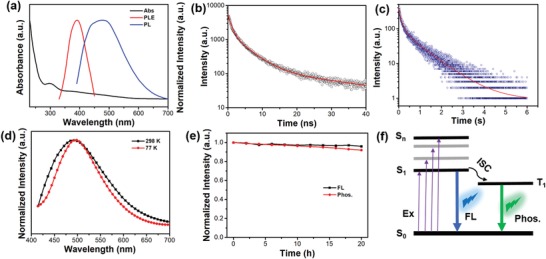
a) Normalized optical absorption (black line), PLE (red line), and PL (blue line) spectra of the SW‐CPDs, respectively. The PLE spectrum is obtained by monitoring the PL peak at ≈436 nm. The PL spectrum is recorded upon excitation at ≈375 nm. b,c) Time‐resolved PL spectra by monitoring the PL peaks at 436 nm (b) and 495 nm (c), respectively. d) Phosphorescence spectra of the SW‐CPDs measured at 298 and 77 K upon excitation at 375 nm, respectively. e) Photostability of SW‐CPDs under continuous illumination with an UV (365 nm) lamp for 20 h. f) Illustration of FL and phosphorescence (phos.) processes of SW‐CPDs.

Transmission electron microscope (TEM) measurements show that the SW‐CPDs are well‐dispersed quasi‐spherical nanoparticles with an average size of ≈3.4 nm (**Figure** **3**a and Figure S5 in the Supporting Information). The high‐resolution TEM (HRTEM) image indicates that the SW‐CPDs are amorphous structures without lattice fringes, which is inconsistent with the reported CPDs (inset of Figure 3a).^28^ The atomic force microscope (AFM) image shows that the topographic height of the SW‐CPDs are homogenously distributed in the range of 1.0–2.0 nm (Figure S6 in the Supporting Information), indicating a high morphological uniformity. The X‐ray diffraction (XRD) pattern displays a broad band centered at 21.5° (Figure S7 in the Supporting Information), which confirms the amorphous nature of SW‐CPDs resulting from covalently crosslinked frameworks. Raman spectrum of SW‐CPDs shows a characteristic D band at 1365 cm^−1^ and a G band at 1579 cm^−1^ (Figure S8 in the Supporting Information). The intensity ratio *I*_G_/*I*_D_ is ≈1.16, lower than the crystalline CDs,^16^ which further confirms the amorphous nature of the SW‐CPDs.

**Figure 3:**
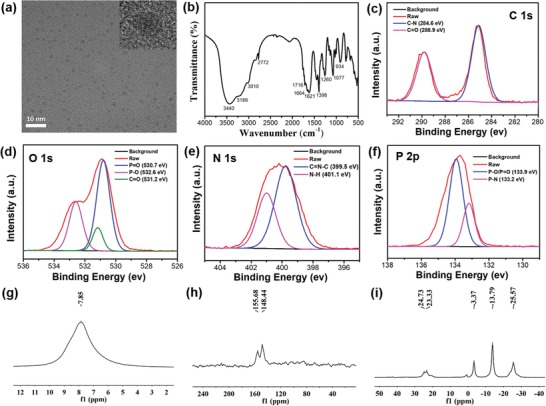
a) TEM image of SW‐CPDs. The inset is the HRTEM image of an individual CD. b) FT‐IR spectrum of SW‐CPDs. c–f) High‐resolution XPS results for the C 1s (c), O 1s (d), N 1s (e), and P 2p (f) spectra of SW‐CPDs, respectively. g–i) NMR spectra for ^1^H (g), ^13^C (h), and ^31^P (i) of SW‐CPDs, respectively.

The chemical compositions of the SW‐CPDs are characterized by Fourier transform infrared (FT‐IR) spectroscopy. As shown in Figure 3b, the strong stretching vibration bands of O—H and N—H are observed at 3440 and 3186 cm^−1^, respectively.^29^ The peak located at 1621 cm^−1^ corresponds to the bending vibration of N—H. The strong stretching vibration bands of C=N, C=O, and C—N are observed at 1664, 1716, and 1398 cm^−1^, respectively.^30^ The peaks located at 1260, 1077, and 934 cm^−1^ should be caused by P=O, P—O, and P—N vibrations.^31^, ^32^ X‐ray photoelectron spectroscopy (XPS) analysis suggests that the SW‐CPDs are mainly composed of C, O, N, and P elements with atomic ratios to be 22.66%, 24.91%, 40.78%, and 11.65%, respectively, indicating heavy doping contents of N and P (Figure S9 in the Supporting Information). The C 1s band can be deconvoluted into two peaks at 284.6 and 288.9 eV, corresponding to C—N and C—O (Figure 3c). The O 1s band contains three peaks at 530.7, 531.2, and 532.6 eV due to =O, C=O, and C—O, respectively (Figure 3d).^33^ As shown in Figure 3e, the N 1s analysis reveals the presence of C=N—C (399.5 eV) and N—H (401.1 eV). The P 2p band is fitted with two peaks at binding energies of 133.2 and 133.9 eV, which can be assigned to P—N and P=O, respectively (Figure 3f).^26^ To further confirm the above FT‐IR and XPS analyses, solid‐state NMR spectra of the SW‐CPDs are measured. In the ^1^H NMR spectrum (Figure 3g), only a single active H signal is observed in the range of 7.0–8.0 ppm, corresponding to N—H and —OH. The ^13^C NMR spectrum shows a resonance in the range of 145–160 ppm, indicating the presence of carbon atoms in imide functional groups of the SW‐CPDs (Figure 3h). Moreover, the peaks observed between 0 and 30 ppm in the ^31^P NMR spectrum are associated with organic phosphate, confirming the presence of P=O and P—O species in the SW‐CPDs (Figure 3i).

Taken together, the origins of fluorescence/phosphorescence dual emission from the SW‐CPDs may be tentatively proposed as follows. We deduce that the selected precursors are the key to forming SW‐CPDs. Urea could serve both as a carbon source for carbonization and a nitrogen source for doping, while phosphoric acid could serve as a catalyst for dehydration, deamination, and a crosslink agent for polymerization. A polymerization reaction is ready to take place in a heating environment by combining the ‐NH_2_ group in urea and the ‐OH group in phosphoric acid to form a long‐chain polymer, as illustrated in Figure S10 (Supporting Information). Subsequently, deamination and dehydration reactions between long‐chain polymers occur, generating SW‐CPDs with covalently crosslinked frameworks. According to the above formation process, the PL mechanism of the SW‐CPDs can be attributed to crosslink‐enhanced emission effect,^24^ because abundant sub‐fluorophores are present in SW‐CPDs, such as —OH, —NH_2_, C=O, C=N, N—P, C—N=C, and C—N—P functional groups. The heavy doping of N and P elements benefits in the n→π* transition to produce phosphorescence with a high QY because of the small energy gap between S_1_ and T_1_. In addition, the covalently crosslinked framework of SW‐CPDs can self‐immobilize the excited triplet states in the SW‐CPDs to suppress quenching of phosphorescence by oxygen at room temperature. Therefore, the white light emission composed of fluorescence/phosphorescence dual emissions from SW‐CPDs is supposed to be originated from the intertwined polymeric framework doped with N and P elements.

Theory calculations were performed to support the proposal for white light emission of SW‐CPDs. The computation model is built by using a small repeated structural unit from the presumed structure of SW‐CPDs (Figures S11 and S12a in the Supporting Information). The molecular frontier orbital amplitude plots, including highest occupied molecular orbital, lowest unoccupied molecular orbital, FL, and phosphorescence, are summarized in Figures S9b,c and S13 (Supporting Information). The calculated FL and phosphorescence spectra are approximately consistent with the experimental data. The calculated Δ*E*_ST_ between S_1_ and T_1_ is 0.24 eV, which is close to the experimental value of 0.09 eV (Figure S14 in the Supporting Information). The small magnitude of Δ*E*_ST_ indicates that ISC process can readily occur between S_1_ and T_1_ (S_1_ → T_1_).

The high QY and excellent photostability as well as low‐cost and large‐scale fabrication of SW‐CPDs strongly suggest that the SW‐CPDs hold potential in constituting high‐performance WLED. Given that the PLE spectrum shows a maximum at ≈375 nm, the SW‐CPDs are compatible with commercial 370 nm UV‐LED chips to fabricate WLEDs. As a preliminary demonstration, the SW‐CPDs are uniformly dispersed into silicone and then coated on the UV‐LED chip to form a WLED. The electroluminescence (EL) spectrum of the as‐fabricated WLED is shown in **Figure** **4**a. Two distinct peaks are clearly observed: the one at 430 nm corresponding to the blue FL and another at 503 nm originating from the green phosphorescence. The WLED emits bright cool white light with correlated color temperature (CCT) of 8756 K and CIE color coordinates of (0.268, 0.346) (inset of Figure 4a,b and Video S2 in the Supporting Information). In addition, the WLED features a high CRI of 85.3 and a high luminous efficacy of 18.7 lm W^−1^. We notice that the blue‐LED pumping usually gives a relatively higher luminous efficacy due to the fact that the light emitted from the blue‐LED chip will directly constitute an important spectral portion of the white‐light spectrum from CDs‐based WLEDs (Table S2, Supporting Information).^34^, ^35^ On the other hand, our WLEDs exhibit a high luminous efficacy among UV‐based devices and even comparable to that exhibited in our recently developed WLEDs based on blue‐, green‐, and red‐emitting CDs with apparently higher PLQY (Table S2, Supporting Information).^16^, ^23^, ^36^, ^37^ The EL intensity of the WLED maintains 92.5% of the initial value after 144 h of continuous operation at 20 mA (Figure S15 in the Supporting Information). The degradation of the EL signal mainly occurs during the first 5 h and then the EL performance keeps stable (Figure S16 in the Supporting Information).

**Figure 4:**
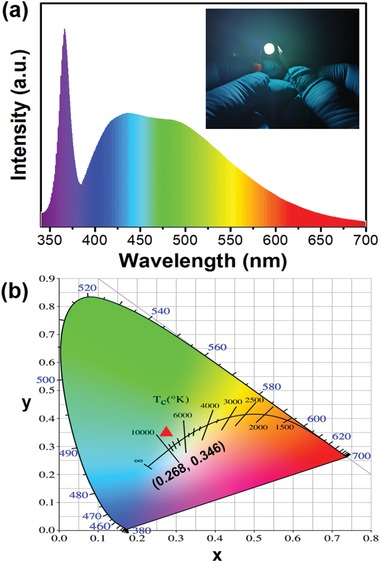
a) EL spectrum and b) CIE color coordinate of the WLED based on SW‐CPDs. The inset in (a) is a photograph of the WLED driven by a current of 20 mA.

In conclusion, we have successfully devised a method to synthesize gram‐scale SW‐CPDs with an overall QY up to 41%, which is the highest value recorded for solid‐state fluorescent CDs. The synthesis is based on a one‐step process involving polymerization, deamination, and dehydration reactions of urea and phosphoric acid in an aqueous environment. The obtained SW‐CPDs exhibit white light with dual components consisting of simultaneous FL (S_1_→S_0_) and phosphorescence (T_1_→S_0_). Detailed structural and optical characterizations as well as elaborate theoretical calculations suggest that intertwined polymeric framework doped with N and P accounts for the observed white light emission. A UV‐pumped WLED is fabricated by coating SW‐CPDs on a UV‐LED chip, which shows favorable white light characteristics with the CIE coordinates, CCT and CRI of (0.268, 0.346), 8756 K ,and 85.3 and exhibits excellent device stability after continuously working for 144 h. The luminous efficacy of the optimized WLED is ≈18.7 lm W^−1^, which is high among UV‐excited CPDs‐based WLEDs and comparable to some of rare‐earth phosphors‐based WLEDs. This work suggests that rationally designing the framework of CPDs with functional groups is of significance to achieve large‐scale SW‐CPDs to meet various purposes. We anticipate that future endeavors will further improve the performance of SW‐CPDs to be suitable for lighting applications in WLEDs.

## Experimental Section

##### Materials

Urea (99.5%) was bought from Aladdin Chemicals Co. Ltd. (Shanghai, China). Phosphoric acid was purchased from Sinopharm Chemical Reagent Co. Ltd. (Shanghai, China). All chemicals were used as received without further purification unless otherwise specified. Deionized water was used throughout this study.

##### Characterization Method

A JEOL JEM 2100 TEM was used to examine the morphologies of SW‐CPDs. AFM image was taken with MultiMode V SPM (VEECO). The XRD pattern was measured using Cu‐Kα radiation using a PANalytical X'Pert Pro MPD powder diffractometer. Optical absorption spectra were recorded on an UV‐2600 spectrophotometer. The PL spectra and time‐resolved PL decay data were obtained using a spectrometer (FLS980) from Edinburgh Instruments. The absolute overall QYs and phosphorescence QYs were obtained using an Edinburgh FLS980 FL spectrophotometer equipped with a xenon arc lamp (Xe900) and a microsecond flashlamp (µF900), and an integrating sphere, respectively. The photographs were taken with a camera (Nikon, D7200) under UV lamp illumination working at 365 nm (UV lamp: SPECTROLINE, ENF‐280C/FBE, 8 W). The FT‐IR spectrum was measured using a Nicolet 380 spectrograph. The XPS spectrum was measured with an ESCALab220i‐XL electron spectrometer from VG Scientific using 300 W Al Kα radiation. The Raman spectrum was measured using laser confocal Micro‐Raman spectroscopy (LabRAM Aramis). NMR spectra were recorded in Bruker DRX500.

##### Synthesis of SW‐CPDs

Urea (1.5 g) and phosphoric acid (0.2 mL) were dissolved in deionized water (10 mL) to form a clear solution. The resultant solution was transferred to a beaker (50 mL), heated at 200 °C for 3.0 h, and then naturally cooled down to room temperature. Pale yellow powders were formed during the cooling process. Finally, the SW‐CPDs were obtained by washing with deionized water three times and freeze‐drying, respectively.

##### Fabrication of WLEDs

An UV‐LED chip (LUMEX‐SSL‐LXTO46UV1C) with the peak emission wavelength centered at 370 nm was used for the fabrication of WLED. A certain amount of SW‐CPDs was mixed with silicone thoroughly and then the resultant phosphor–silicone mixture was coated on the surface of the UV‐LED chip to produce WLED. The optoelectronic properties of the fabricated devices were measured by an integrating sphere spectroradiometer system (LHS‐1000, Everfine).

## Conflict of Interest

The authors declare no conflict of interest.

## Supporting information

Supporting Information

Supplemental Video 1

Supplemental Video 2
