# Supplementary material for: Gram‐Scale Synthesis of 41% Efficient Single‐Component White‐Light‐Emissive Carbonized Polymer Dots with Hybrid Fluorescence/Phosphorescence for White Light‐Emitting Diodes
Source: Adv Sci (Weinh). 2020 Jan 16;7(4):1902688. doi: 10.1002/advs.201902688 (PMC7029630; doi:10.1002/advs.201902688)
Supplement: Supplementary file 1 — Supporting Information [file ADVS-7-1902688-s001.pdf]

Copyright WILEY-VCH Verlag GmbH & Co. KGaA, 69469 Weinheim, Germany, 2020.

ADVANCED SCIENCE
Open Access

# Supporting Information

for *Adv. Sci.,* DOI: 10.1002/advs.201902688

# Gram-Scale Synthesis of 41% Efficient Single-Component White-Light-Emissive Carbonized Polymer Dots with Hybrid Fluorescence/Phosphorescence for White Light-Emitting Diodes

*Zifei Wang,* Yang Liu, Shijie Zhen, Xiaoxi Li, Weiguang Zhang, Xun Sun, Baoyuan Xu, Xue Wang, Zhenhua Gao, and Xiangeng Meng**

Copyright WILEY-VCH Verlag GmbH & Co. KGaA, 69469 Weinheim, Germany, 2019.

Supporting Information

# Gram-Scale Synthesis of 41% Efficient Single-Component White Light Emissive Carbonized Polymer Dots with Hybrid Fluorescence/Phosphorescence for White Light-Emitting Diodes

*Zifei Wang,* Yang Liu, Shijie Zhen, Xiaoxi Li, Weiguang Zhang, Xun Sun, Baoyuan Xu, Xue Wang, Zhenhua Gao and Xiangeng Meng**

Dr. Z. F. Wang, Y. Liu, X. X. Li, W. G. Zhang, X. Sun, B. Y. Xu, Dr. X. Wang, Dr. Z. H. Gao, Prof. X. G. Meng
School of Materials Science & Engineering, Qilu University of echnology (Shandong Academy of Sciences), Jinan, 250300, China
E-mail: wangzifei2013@126.com; mengxiangeng@gmail.com
Dr. S. J. Zhen
State Key Laboratory of Luminescent Materials and Devices, South hina University of Technology, Guangzhou, 510640, China

**Preparation of the TEM sample**

The SW-CPDs (50 mg) was dissolved in 10 mL deionized water by sonication. Then the SW-CPDs solution was subjected to dialysis for a week in order to obtain the pure SW-CPDs. Finally, TEM sample was prepared by drop casting diluted SW-CPDs solution on a carbon-coated copper grid and examined in JEOL JEM 2100 TEM.

**QY measurements of phosphorescence**

An absolute method, using FLS980 fluorescence spectrometer equipped with an integrating sphere and door-control device, was employed to determine the QY of SW-CPDs. We conducted the test light from a microsecond flash-lamp (μF900) to the sphere. The QY was determined by the sphere when the delay time is set to 1 ms using door-control device. The SW-CPDs powder was placed in a quarts cuvette with a light path to measure its QY, while $Al_2O_3$ powders were used as a blank sample for the reference measurement. All acquired data was be corrected by a file from main unit automaticity.

**Theoretical calculations**

The ground-state geometries were optimized using the density functional theory (DFT) with B3LYP hybrid function at the basis set level of 6-31G (d, p). The calculations of the electronic structure were performed with the Gaussian 09 program using DFT and time-dependent density functional theory (TD DFT), respectively.

**Table S1** The production yield of SW-CPDs.

| | Raw materials (g) | Resultant SW-CPDs | Production yield (%) | Average (%) |
|---|---|---|---|---|
| 1 | 1.7 | 1.28 | 75.3% | |
| 2 | 1.7 | 1.39 | 81.7% | 81% |
| 3 | 1.7 | 1.46 | 85.9% | |

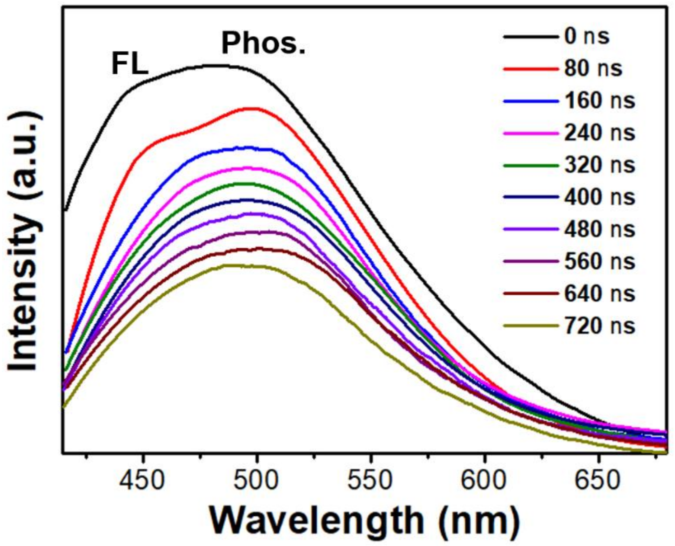


**Figure S1.** Time-dependent PL spectra of SW-CPDs.

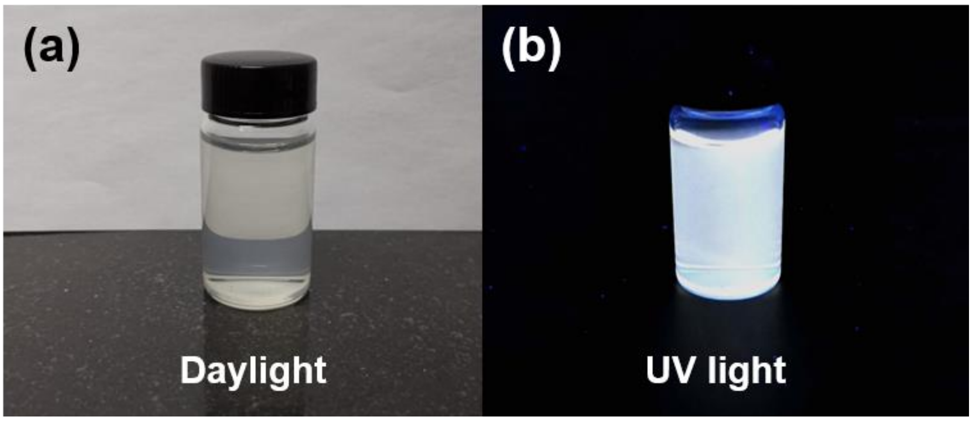


**Figure S2.** Photographs of SW-CPDs captured under daylight (a) and 365 nm UV lamp illumination (b).

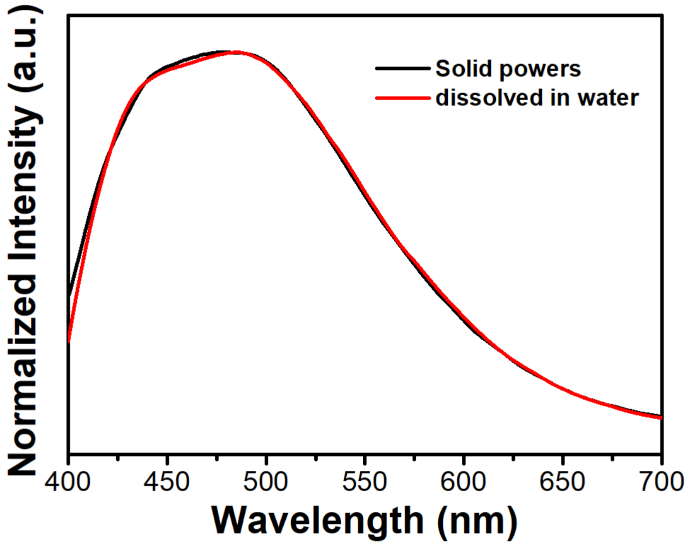


**Fiure S3.** PL spectra of solid-state SW-CPDs (black curve) and the SW-CPDs suspended in water (red curve). The excitation wavelength is 375 nm for both PL spectra.

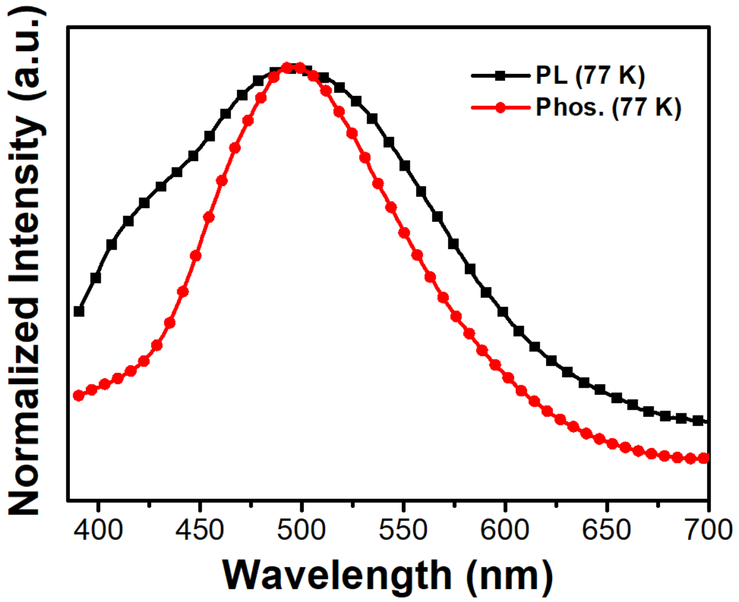


**Figure S4.** PL and phosphorescence (phos.) spectra of the SW-CPDs recorded at 77 K under the excitation wavelength at 370 nm.

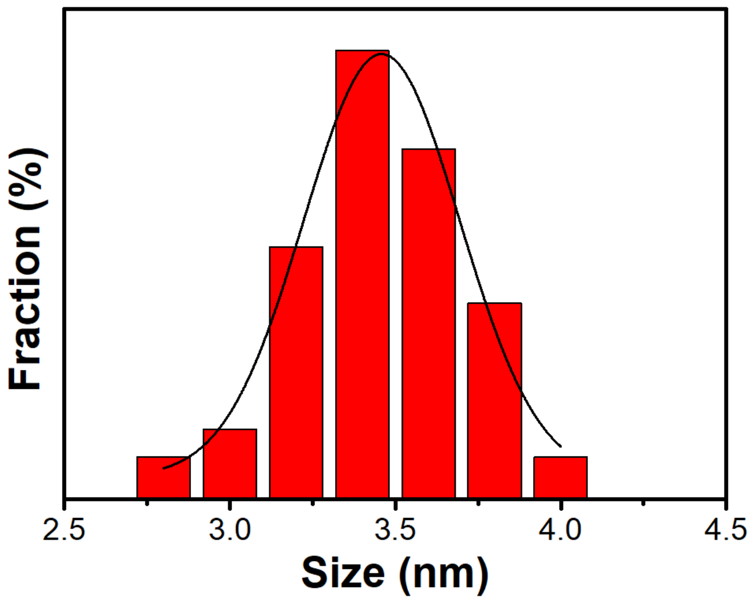


**Figure S5.** Size distribution of SW-CPDs.

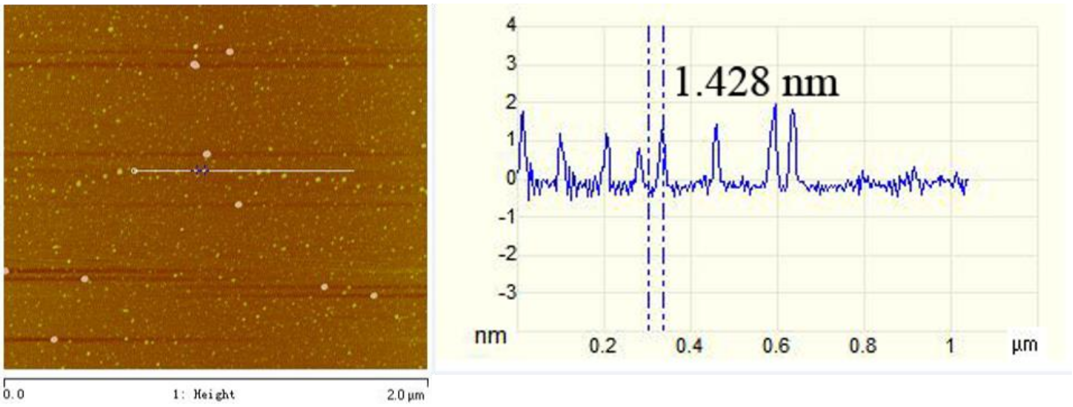


**Figure S6.** AFM image and height profiles of SW-CPDs.

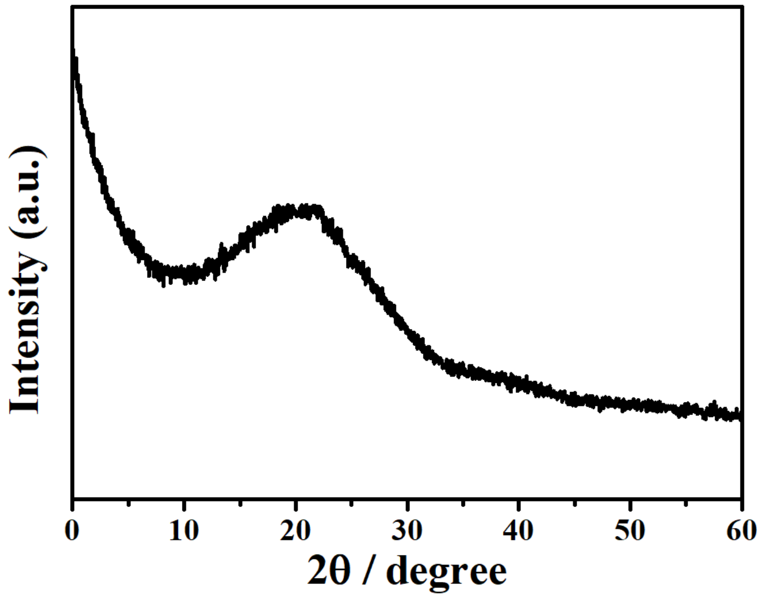


**Figure S7.** XRD pattern of SW-CPDs

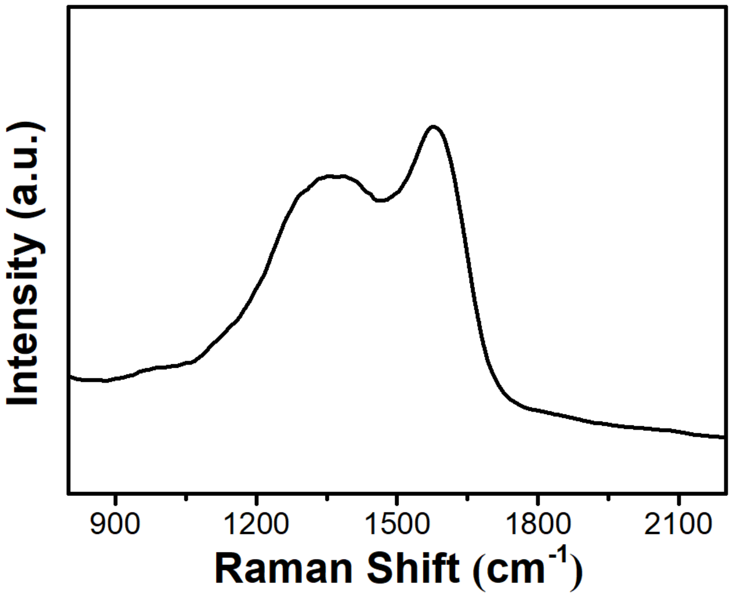


**Figure S8.** Raman spectrum of SW-CPDs.

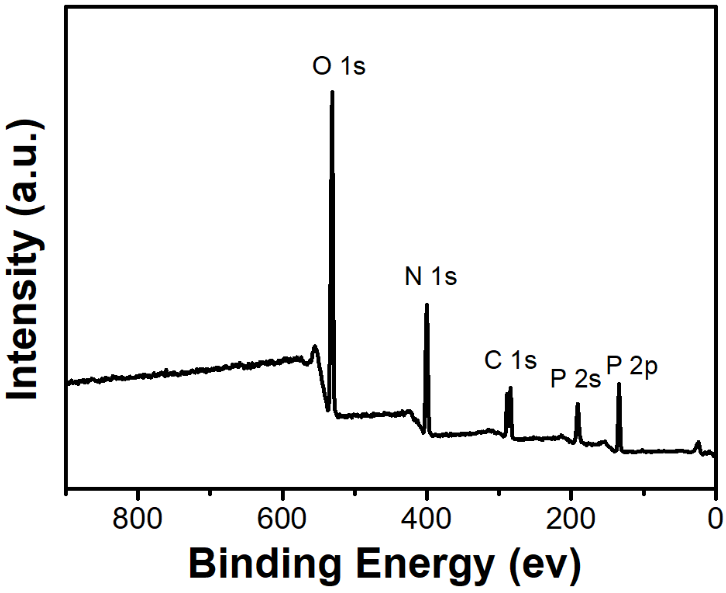


**Figure S9.** XPS survey spectrum of SW-CPDs.

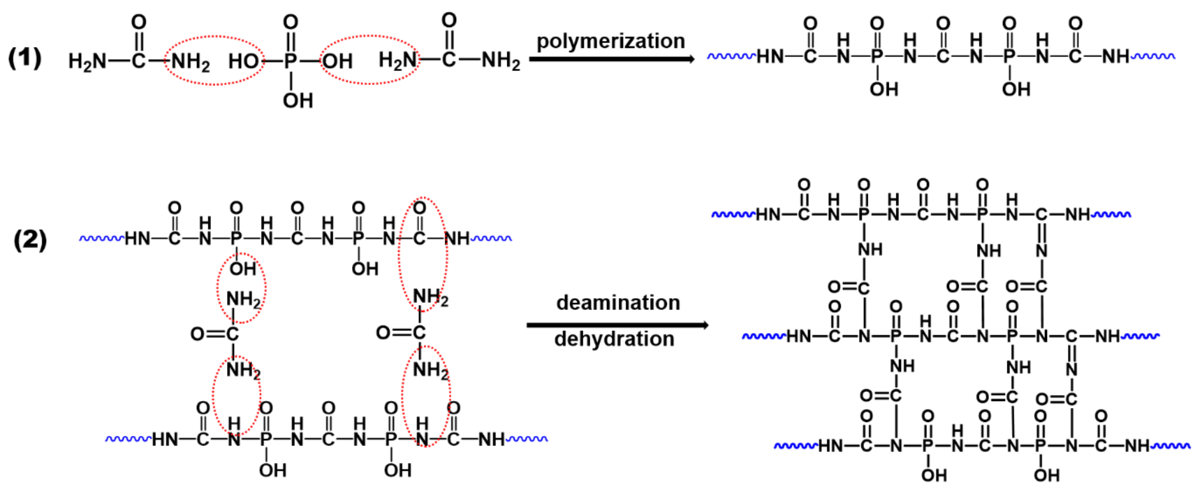


**Figure S10.** Illustration of the growth mechanism of SW-CPDs.

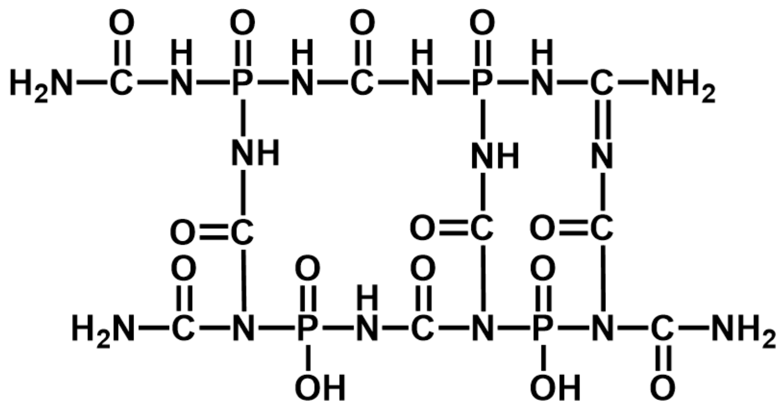

**Figure S11.** Small structure unit of SW-CPDs utilized for theoretical calculation.

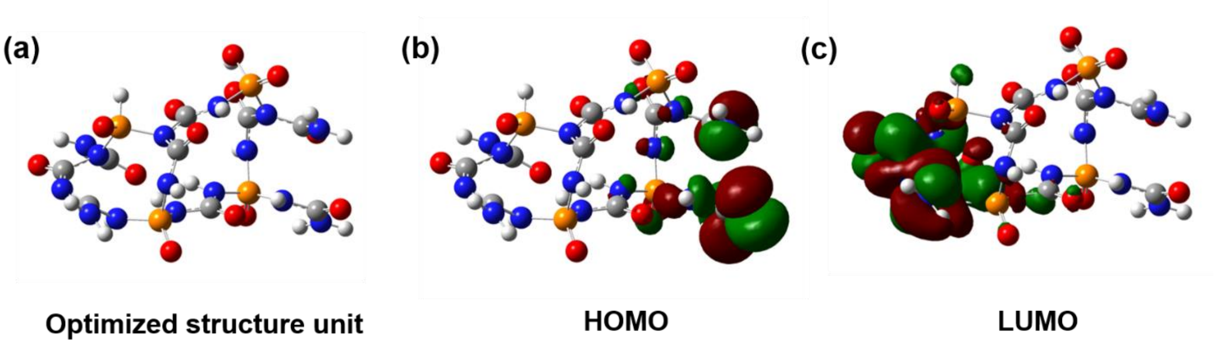


**Figure S12.** Time-dependent density functional theory calculation results. (a) Optimized structure unit of SW-CPDs for theoretical calculation. The calculated HOMO (b) and LUMO (c) of SW-CPDs.

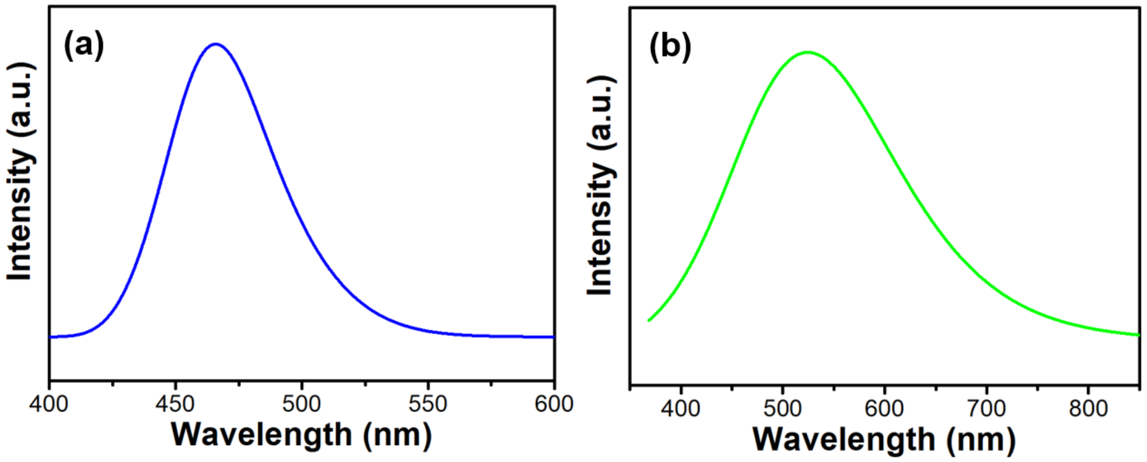


**Figure S13.** Calculated FL (a) and phosphorescence (b) spectra of SW-CPDs.

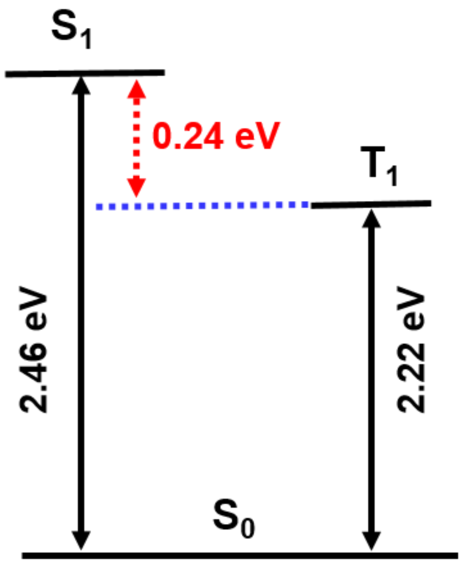


**Figure S14.** Calculated energy levels between $S_1$ and $T_1$ of SW-CPDs.

**Table S2.** Comparison of luminous efficacy of WLEDs based on CDs and rare-earth phosphors operated at 20 mA drive current.

| Excitation source | Luminescent materials | Luminous efficacy (lm $W^{-1}$) | References |
| --- | --- | --- | --- |
| blue LED chip | green-yellow emissive CDs | 28 | [34] |
| blue LED chip | green and red emissive CDs | 86.5 | [35] |
| UV LED chip | blue, green and red emissive CDs | 18.8 | [16] |
| UV LED chip | white emissive CDs | 5.65 | [23] |
| UV LED chip | blue and orange emissive CDs | 14.8 | [36] |
| UV LED chip | $Al_{1-x}Si_xC_xN_{1-x}:Eu^{2+}$/LuAG:$Ce^{3+}$/ (Sr,Ca)$AlSiN_3:Eu^{2+}$ | 7.16 | [37] |
| UV LED chip | SW-CPDs | 18.7 | This work |

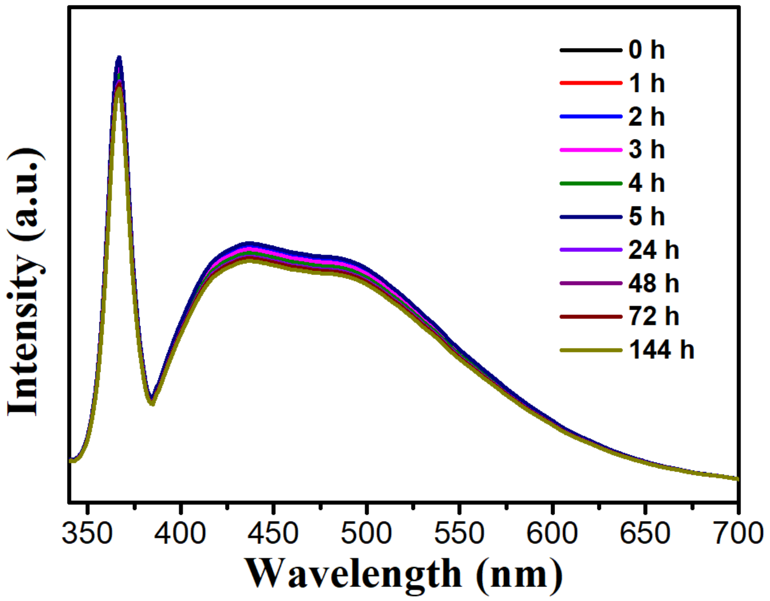


**Figure S15.** EL spectra of the WLED recorded under different operating time intervals.

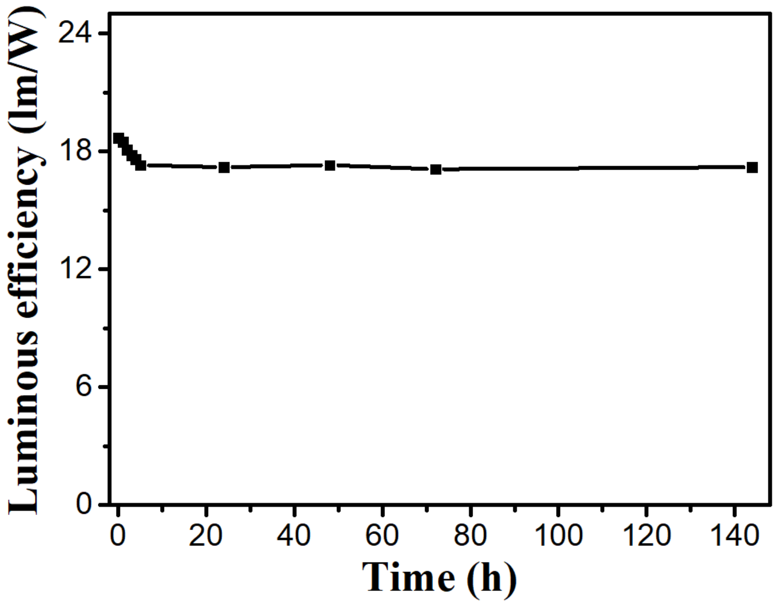


**Figure S16**. Luminous efficacy of the WLED recorded under different operating time intervals.
